# Intra- and Inter-Subject Perspectives on the Detection of Focal Onset Motor Seizures in Epilepsy Patients

**DOI:** 10.3390/s22093318

**Published:** 2022-04-26

**Authors:** Sebastian Böttcher, Elisa Bruno, Nino Epitashvili, Matthias Dümpelmann, Nicolas Zabler, Martin Glasstetter, Valentina Ticcinelli, Sarah Thorpe, Simon Lees, Kristof Van Laerhoven, Mark P. Richardson, Andreas Schulze-Bonhage

**Affiliations:** 1 Epilepsy Center, Department of Neurosurgery, Medical Center—University of Freiburg, 79106 Freiburg im Breisgau, Germany; nino.epitashvili@uniklinik-freiburg.de (N.E.); matthias.duempelmann@uniklinik-freiburg.de (M.D.); nicolas.zabler@uniklinik-freiburg.de (N.Z.); martin.glasstetter@uniklinik-freiburg.de (M.G.); andreas.schulze-bonhage@uniklinik-freiburg.de (A.S.-B.); 2 Ubiquitous Computing, Department of Electrical Engineering and Computer Science, University of Siegen, 57076 Siegen, Germany; kvl@eti.uni-siegen.de; 3 The RADAR-CNS Consortium, London WC2R 2LS, UK; elisa.bruno@kcl.ac.uk (E.B.); valentina.ticcinelli@ucb.com (V.T.); sarahthorpe84@gmail.com (S.T.); simonintheasylum@gmail.com (S.L.); mark.richardson@kcl.ac.uk (M.P.R.); 4 Division of Neuroscience, Institute of Psychiatry, Psychology & Neuroscience, King’s College London, London SE5 9RT, UK; 5 UCB Pharma, 1070 Anderlecht, Belgium; 6 The RADAR-CNS Patient Advisory Board, King’s College London, London WC2R 2LS, UK

**Keywords:** wearables, epilepsy, seizure detection, multimodal, mHealth, eHealth, mobile health, digital health

## Abstract

Focal onset epileptic seizures are highly heterogeneous in their clinical manifestations, and a robust seizure detection across patient cohorts has to date not been achieved. Here, we assess and discuss the potential of supervised machine learning models for the detection of focal onset motor seizures by means of a wrist-worn wearable device, both in a personalized context as well as across patients. Wearable data were recorded in-hospital from patients with epilepsy at two epilepsy centers. Accelerometry, electrodermal activity, and blood volume pulse data were processed and features for each of the biosignal modalities were calculated. Following a leave-one-out approach, a gradient tree boosting machine learning model was optimized and tested in an intra-subject and inter-subject evaluation. In total, 20 seizures from 9 patients were included and we report sensitivities of 67% to 100% and false alarm rates of down to 0.85 per 24 h in the individualized assessment. Conversely, for an inter-subject seizure detection methodology tested on an out-of-sample data set, an optimized model could only achieve a sensitivity of 75% at a false alarm rate of 13.4 per 24 h. We demonstrate that robustly detecting focal onset motor seizures with tonic or clonic movements from wearable data may be possible for individuals, depending on specific seizure manifestations.

## 1. Introduction

With a worldwide incidence of up to 100 per 100,000 per year, epilepsy is one of the most prevalent neurological disorders, affecting over 70 million people worldwide [1]. Epilepsy manifests in a multitude of different symptoms with varying severities generally denoted as epileptic seizures [2], and the current gold standard in diagnosis and seizure monitoring is in-hospital video-electroencephalography (vEEG). However, this diagnostic tool, while being accurate and widely used for diagnosis and determination of treatment, is only practicable in relatively short-term applications at a hospital or special epilepsy monitoring unit [3]. At home or in their daily life, patients with epilepsy cannot feasibly be monitored in such a way and with the same benefit. Yet, seizure tracking in an ultra-long-term context at home is needed, both as an automatic alarm to potentially alert carers in the event of a seizure, and as a tool for monitoring and forecasting the disease trajectory [4,5]. Thus, different technologies and methodologies of seizure monitoring need to be employed, and wearables such as smartwatches and fitness trackers in combination with machine learning may fill this gap while being easily available for a wider audience.

Epileptic seizures are defined by a period of abnormal neuronal activity in the brain, and are generally divided into two main groups by their neurological onset [2]. Seizures with an early bilateral involvement are called generalized seizures, while seizures with just a single point of onset are denoted as focal onset seizures, but epileptic activity can propagate across the brain resulting in “focal to bilateral” seizures with characteristic motor manifestations. Bilateral tonic–clonic seizures have been assessed in numerous studies as to the viability of wearables for the detection during recent years, and detection has been demonstrated to be feasible in multiple retrospective studies [6,7,8,9,10,11,12,13,14]. Conversely, focal seizures in general are still a relatively unexplored field with respect to wearable non-EEG detection [15,16,17,18,19,20]. Symptoms and manifestations of these seizures are much more heterogeneous as compared to those of bilateral tonic–clonic seizures, with some barely or not at all captured by typical wearable biosignal modalities, such as accelerometry (ACC), electrodermal activity (EDA), or photoplethysmography (PPG).

Focal seizures can be roughly divided into two categories: those with motor and those without motor manifestations. Non-motor symptoms, that is, those without involuntary movement of the body, may include partial loss of awareness or consciousness, cognitive impairment, or emotional or sensory symptoms. Motor symptoms, on the other hand, can include tonic or clonic movements of the limbs or body in general, hyperkinetic movements, or automatisms. A single epileptic seizure can thereby be composed of multiple types of manifestations, in parallel or sequentially.

This work highlights difficulties in the detection of focal onset epileptic seizures, specifically those with focal tonic or clonic motor symptoms but without bilateral propagation, from biosignal data captured by wearable devices. A data set consisting of multimodal data from a wrist-worn wearable was recorded from patients with epilepsy during their in-hospital stay at an epilepsy monitoring unit (EMU). Classical supervised machine learning is applied in order to assess the utility of this kind of data for the detection of seizures, in the context of an automated seizure diary. As written diaries created by the patients themselves have been demonstrated to be very inaccurate and often severely undercount seizures even for convulsive seizures [21,22,23], an automated diary tool implementing an objective seizure identification and quantification is needed, for example, as a basis for treatment decisions made by epileptologists. In the supervised methodology employed here, data are first labeled, based on parallel vEEG monitoring, as “seizure” or not “seizure”, and then processed into meaningful features and given to the machine learning model for training. Thus, the trained model can then be used to automatically classify new data. Specifically, in this study we employ a gradient tree boosting machine as the seizure detection model. To evaluate such an approach, and subsequently also determine the application in a real-world system, two procedures can be applied: intra-subject or inter-subject evaluation. The intra-subject evaluation focuses on the performance of the methodology when applied to data from a single patient, while the inter-subject evaluation assesses the performance over multiple patients with potentially different types of epilepsy and seizure manifestations. The former requires multiple seizures recorded per subject and will produce individualized models tailored to a single patient, while the latter requires seizures recorded from multiple different participants and will give inter-subject models, to be used over wider populations. Here, we aim to determine which of these approaches may work best for focal motor seizures going forward, giving guidance for the design of future studies in the field. Following the study classification suggested by Beniczky and Ryvlin in 2018 [24], the work presented here could be classified as a phase 1 retrospective proof-of-principle study. The main contribution of this work is the evaluation of supervised machine learning methodologies on focal onset epileptic motor seizures in a data set recorded from a non-EEG wearable device. A comparison between two evaluation approaches, intra- and inter-subject, provides additional context and facilitates recommendations towards future studies in the field.

## 2. Materials and Methods

### 2.1. Data Set

**Figure A1:**
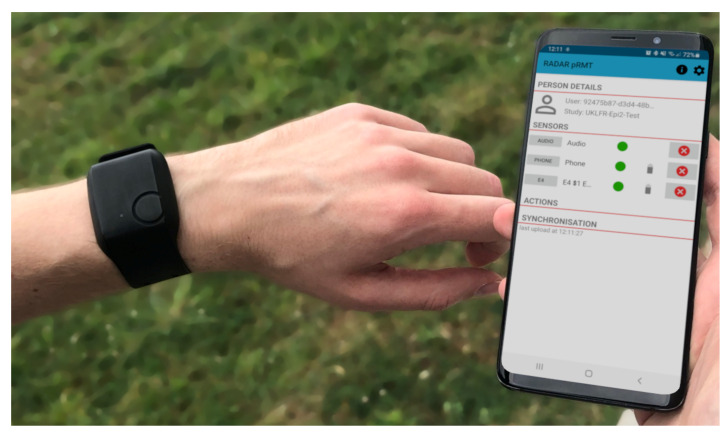
The Empatica E4 wrist-worn wearable device used in this study (**left**), and the Android phone application that connects to the wearable via Bluetooth and records the data stream (**right**).

Data from wearable devices was recorded from a total of 243 patients with epilepsy across two EMUs in the period between July 2017 and February 2020. Both at the neurophysiological department of King’s College Hospital, London (KCL, 71/243), and at the Epilepsy Center, Medical Center—University of Freiburg (UKF, 172/243), patients in the age range of 7 to 80 with a diagnosis of epilepsy were recruited sequentially as part of their standard clinical epilepsy care, for example, in the course of standard presurgical evaluation. Patients with predominantly (suspected) psychogenic non-epileptic seizures or other involuntary movements were not included in the study. As part of their stay in the EMU study, participants may have had seizures provoked, for example, by temporary reduction of their anti-epileptic medication or through other means, such as sleep deprivation or hyperventilation techniques. The vEEG data was retrospectively reviewed and labeled by clinical experts (E.B., N.E.). Primarily, they marked seizure type and semiologies, electrographic and clinical onset and offset, and other meta-data including state of vigilance and body position at seizure onset. While participants wore different kinds of wearable devices, and sometimes more than one in parallel, the retrospective study presented here only includes data from the wrist-worn Empatica E4 (Figure A1; Empatica Inc., Boston MA, USA). It is a research-grade device designed specifically for epilepsy seizure detection recording 3-axis ACC at a sample rate of 32 Hz, EDA at 4 Hz, skin temperature at 4 Hz, and PPG at 64 Hz, the latter of which was internally pre-processed into a blood volume pulse (BVP) signal. The wearable has a European CE class IIa certification as a medical device. The data recording mode used in this study was the online Bluetooth streaming mode. Battery life could range between 12 h to 48 h depending on the condition of the battery. Participants were given two devices, such that one would always be recording while the other was charging. The study and recording procedures are further described in Bruno et al. 2021 [25] and Ranjan et al. 2019 [26]. As part of the study recruitment, all participants gave written informed consent, and the study protocols and consent forms were approved by the local ethics committees (London Fulham Research Ethics Committee—16/LO/2209; Ethics Committee at the University of Freiburg—538/16).

### 2.2. Feature Set

**Figure 1:**
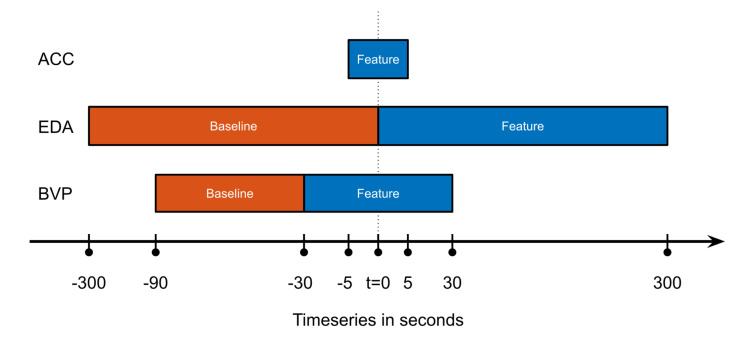
Overview of how the feature and baseline windows were chosen, for the three different groups of features by modality. This calculation would result in one feature vector, for the next the windows would all be shifted by an interval of T = 2 s to the right. Abscissa not to scale.

To facilitate the detection of focal motor seizures in this data set of non-EEG wearable data, a selection of derived features are calculated from each of the three raw data modalities. These features are chosen to meaningfully represent the changes in the signal between ictal (seizure) and inter-ictal (non-seizure) phases. Each feature vector is calculated consecutively from the raw time series data at a constant interval of two seconds, regardless of the actual length of the feature window (see Figure 1). The choice of features in this study was informed primarily by previous research in the field. The following details the feature calculations for the biosignal modalities, ACC, EDA, and BVP.

For the ACC features, a number of parameters calculated from the recurrence plot are used as features. Recurrence plots are a statistical tool to analyze recurrence in time series data [27,28], and have been successfully used in the detection of motor movements from accelerometer data before [7,29,30]. Here, we specifically calculate, from the recurrence quantification analysis, the determinism (percentage of points that form diagonal lines of a minimal length), the Shannon entropy (probability that a line has a certain length), the average diagonal line length, and the recurrence rate (density of recurrence points). All of these values are derived from overlapping data windows of a length of 10 s, centered at each two-second interval.

The EDA features used here are calculated from the skin conductance level (SCL) and the skin conductance response rate (SCRR) [31,32,33,34]. The former is essentially a low-pass filtered version of the original raw EDA signal and thus represents the slower tonic changes in the EDA data. It is represented in the feature set by the difference of area under the curve and maximum between the five minutes before (feature window) and after (baseline) each two-second interval point. Additionally, the SCRR feature is calculated against the baseline in the same way, representing the higher-frequency phasic changes of the EDA signal. The SCRR is calculated as the number of threshold crossings of the first derivative of the EDA signal in the window.

Finally, the BVP raw data (derived device-internally from the PPG sensor) is processed to a heart rate (HR) estimation following the procedure described in Glasstetter et al. 2021 [35]: A peak tracking algorithm was applied to find local minima in the raw time series [36], and the resulting inter-beat-intervals were processed to the HR estimation employing several filters to produce a smooth and meaningful output. This HR estimation as well as a spectral entropy score representing BVP signal quality [35,37] was used as feature values. As the BVP signal is highly sensitive to motion artifacts [38], using a signal quality index like this as a feature for classification follows the principle of regarding artifacts as additional information, instead of discarding them outright. Furthermore, this feature can be observed as a sort of indication for the quality of the model; a model that is highly dependent on the data quality of a signal may not be regarded as a particularly stable model. Additionally, the mean and maximum of the calculated HR feature over a 60-s window are used as features as well, which are baseline-corrected by the difference of values between the feature and baseline window.

An overview of the different feature and baseline windows can be found in Figure 1. A comprehensive listing of the individual features is shown below:

1 Four features calculated from the recurrence plot of the ACC signal in a ten-second window:
  (a) **Determinism**, that is, the percentage of points that form diagonal lines of a minimal length.
  (b) The **Shannon entropy** of the probability that a line has a certain length.
  (c) The **average diagonal line length**.
  (d) **Recurrence rate**, that is, the density of recurrence points.
2 EDA-based features over a five-minute window, minus the same value in the five minutes before the feature window:
  (a) The **area under the curve of the skin conductance level** calculated as the moving mean of the raw EDA signal over a one-minute window.
  (b) The **maximum value of the skin conductance level** calculated as above.
  (c) The **skin conductance response rate** calculated as the number of threshold crossings of the first derivative of the smoothed EDA signal within the window.
3 Heart rate-based features calculated from the BVP signal:
  (a) The **local maximum of the heart rate estimation** in a 60-s window, minus the baseline value from the prior 60-s window.
  (b) The **mean of the heart rate estimation** in a 60-s window, minus the baseline value from the prior 60-s window.
  (c) The **spectral entropy data quality index** of the raw BVP signal, sampled at two-second intervals.
  (d) The **heart rate estimation** calculated from the raw BVP signal, sampled at two-second intervals.

### 2.3. Evaluation

To assess the possibilities of detecting focal epileptic seizures by wearable biosignal data, we investigate two different approaches: intra-subject and inter-subject. The distinction is an important addition to this work, as focal motor seizures have not been investigated to a degree that allows making the choice outright. While an inter-subject approach, i.e., creating models that can detect seizures across a patient population without individual adjustments, is certainly the best possible outcome, the heterogeneity of focal seizures may dictate the use of individualized models. To examine the effect that this might have with the given data set, the evaluation is divided into two parts. First, a subset of participants with at least three seizures recorded is isolated, and the detection model is evaluated per participant in a parameter-optimized leave-one-seizure-out cross-validation. As the data set does not provide data with more than six seizures recorded for a single participant, or with multiple independent recordings of a participant, this is performed without a dedicated test set which is truly “out-of-sample”. Rather, the model is trained with the data of all but one seizure and the respective peri-ictal data of 10 min before and after each seizure. These data are standardized using the z-score method before training, and the normalization parameters (centering mean and scaling standard deviation) are stored. The resulting model is then tested on the remaining participant data and standardized using the previously stored normalization parameters from the training step. This test data includes the complete data set of the participant, including the left-out seizure, but not any of the data used for training the model. Nevertheless, due to the high imbalance between inter-ictal versus ictal phases, the proportion of data between the test and training set is usually far greater than 10:1. This process is repeated such that each seizure of the participant is left out once.

Secondly, the seizure data from all the participants with three or more seizures recorded, selected in the first step, is used to validate the performance on data from all the remaining participants with one or two focal motor seizures recorded. Thereby, the model is first parameter-optimized in a leave-one-participant-out cross-validation on those training participants. Thus, each of the participants in this optimization set is omitted from the model training process once and used as a validation data set. The mean performance scores over the cross-validation runs are then used to determine the optimal parameter combination. In a second evaluation step, the optimized model, now trained with all the peri-ictal seizure data from the training subjects, is then applied to all the data from the test set participants. During the training of this model, the data are again first standardized using the z-score method, and those normalization parameters are then applied to the incoming test data. Overall this results in a model trained and optimized on data from one set of participants, which is then tested on data from another separate set of participants.

### 2.4. Classification Model

The gradient tree boosting machine (GTBM) [39,40] methodology was chosen as the model used to detect ictal states, as it is relatively straightforward in its application and already validated on the same cohort, albeit on data from patients with convulsive seizures [7]. Due to this methodology’s requirement for parameter tuning to achieve good performance, hyperparameter optimization was conducted in both the intra- and inter-subject evaluations, as described above.

For the optimization of the intra-subject model, an optimal parameter combination was found for each of the three included participants by performing a leave-one-seizure-out cross-validation. Thereby, the model was trained on the data of all but one seizure and the respective peri-ictal data, and tested on all remaining data including the left-out seizure for that participant, minus the training data. This was repeated for all seizures, and the performance scores were averaged. This procedure was then repeated for each combination of parameters. For the inter-subject evaluation, a similar procedure was implemented for the three selected participants, but in a leave-one-participant-out manner. The model with the best parameter combination was then tested on out-of-sample data from previously unseen participants.

Four different model parameters were optimized: the learning rate, the maximum number of weak learners, the maximum tree depth per weak learner, and the misclassification cost for false positives. Conversely, the misclassification cost for false negatives was not tuned and kept unweighted, and only one type of boosting was used, namely adaptive boosting for binary classification (“AdaBoost”) [41]. In total, the number of different parameter combinations over which the grid search optimization was performed added up to 600. The best parameter combination was chosen as the one with the highest sensitivity and lowest number of false positives, in that order. In the case of a tie, the parameter combination with a higher learning rate or lower number of trees was chosen as the best one, as it would be computationally more efficient.

To gauge the significance of the various features for the creation of the model, the predictor importance of each of the optimized models was analyzed. Therefore, the importance scores for each of the models resulting from the single cross-validation runs was averaged in the intra-subject leave-one-seizure-out evaluation, resulting in one set of scores per participant included there. Moreover, for the inter-subject leave-one-participant-out evaluation, only the importance scores of the optimal model, trained on all seizures from those intra-subject evaluation participants, were noted. The feature importance was based on decision tree node impurity, using the Gini diversity index and calculated such that the smallest possible value was 0 [42,43]. Thereby, the importance scores for each of the predictors are the averages over all the trained trees in the boosting ensemble for the GTBM model.

### 2.5. Performance Measurement

The main indicators of performance used in this evaluation are the mean sensitivity, false alarm rate (FAR) per 24 h (FAR24) and positive predictive value (PPV). These scores are calculated from the number of overlaps of seizure events in the ground truth and predicted labels. The label data, analogous to the feature data, are stored at two-second intervals, and seizure events here are defined as consecutive intervals of labels classified as a seizure of at least 6 s and at most 10 min. The prediction output of the classification model is furthermore smoothed before this scoring computation, by filling out gaps between seizure labels of at most 30 s, and removing any orphan seizure labels. After this processing of the model output, it is compared to the ground truth and any overlaps of seizure events are counted as true positives, seizure events in the ground truth but not in the predictions are counted as false negatives, and vice versa; seizure events in the predictions that are not present in the ground truth are counted as false positives. Note that the comparisons described above are given a 2-min margin before and after seizure events in the ground truth, wherein overlaps with prediction events still count as true positives. This is performed to account for some of the uncertainty related to seizure manifestations, as well as the in-hospital setting providing a certain degree of nurse intervention after a seizure. True negatives are not counted in this evaluation, as they do not give any more worthwhile information for performance measurement. We also calculated the false alarm rate per night (FARn), that is, during a standard eight-hour night between 23:00 and 07:00. Thereby, we counted how many of the false alarms produced by the model occur during that time period, and divide by the number of hours that were recorded during nights, taking into account any data loss that may have occurred during these hours. The FAR per night is therefore calculated as $\mathrm{FARn}=\mathrm{number}\;\mathrm{of}\;\mathrm{FP}\;\mathrm{during}\;\mathrm{night}\;\cdot \;\frac{\mathrm{hours}\;\mathrm{per}\;\mathrm{night}}{\mathrm{nightly}\;\mathrm{hours}\;\mathrm{recorded}}$.

All data analysis, feature extraction, and performance evaluation was implemented using MATLAB R2021b (MathWorks, Natick, MA, USA).

### 2.6. Data Set Selection

**Figure 2:**
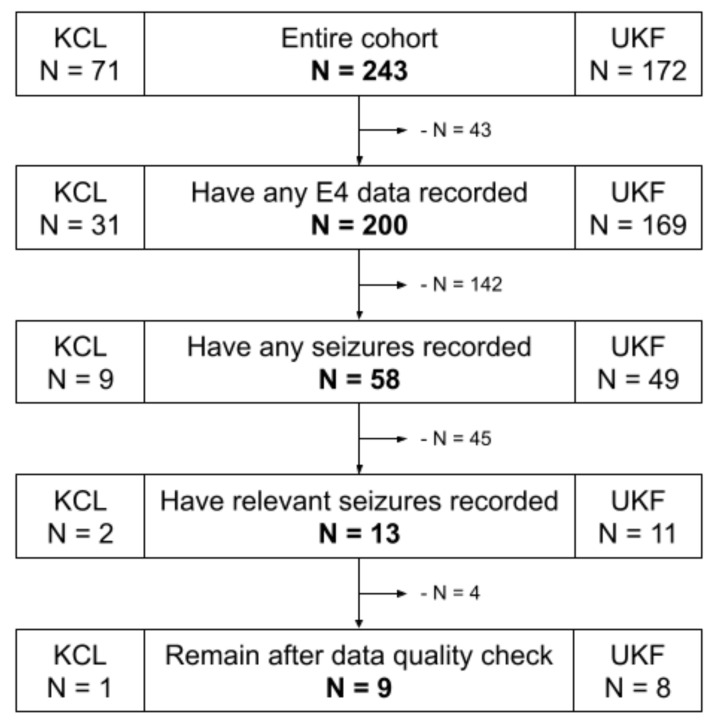
Data set flowchart of the participant selection process. KCL: King’s College London; UKF: University Medical Center Freiburg; E4: Empatica E4 wrist-worn wearable device.

The complete data set of wearable data from 243 patients with epilepsy was filtered to include only data relevant to the premise of this study. First, only data from those participants who had at least one focal seizure recorded that involved tonic or clonic motor manifestations were included. Thereby, these manifestations could co-occur with other seizure manifestations; however, focal to bilateral tonic–clonic seizures were excluded. Moreover, the data set was not filtered further by overlap of symptom location versus device location. Therefore, the data set can, for example, include instances of motor seizures that manifest primarily on the right hand side, but where the wearable device was attached to the left wrist. Excluding these seizure instances would significantly reduce the number of seizures and included participants for the analysis presented here, especially for the inter-subject evaluation, to the point of impracticality. During the study recordings, the Empatica E4 device was used in a Bluetooth streaming mode [25], which unfortunately led to a significant loss of data due to regular problems with connectivity of the wearable device to a base device that stores the data. Thereby, more than 50% of the potential data to be recorded, and correspondingly as many potential seizures, were lost, leading to a significantly reduced number of relevant focal motor seizures recorded for this study. Furthermore, the data set was filtered for total length of recording per participant, where only those recordings with at least 24 h of data were included, and for length of seizures, where only those seizures with a duration between 10 s and 10 min are included. Limiting the duration of seizures excludes very short seizures of just a few seconds, such as myoclonic seizures, and very long seizures, such as status epilepticus. This is performed to exclude outliers and to have defined limits of duration within which to detect potential seizure events. Lastly, the data quality during all remaining seizures was visually checked, and specifically those with bad EDA signal quality were excluded. A poor EDA signal can either be a flat zero-line, indicating loss of contact of the electrodes with the skin, or multiple periods of high rates of amplitude change, indicating a loosely fitting device. The BVP raw signal was specifically not filtered for signal quality, firstly because it would filter out nearly every remaining seizure due to its high susceptibility to motion artifacts, and secondly because the feature set for this evaluation indeed includes a data quality index as a feature itself. Figure 2 visualizes the data set selection process, and Table A2 lists clinical and demographic information of the finally selected participants.

## 3. Results

### 3.1. Data Set and Examples

The resulting data set used for this evaluation thus included 20 relevant seizures from a total of nine study participants. The participants were 44% female (4 of 9) and had a mean age of 45 years (range 9 to 69 years) at study enrollment. Three of these participants had more than two seizures recorded for a total of twelve seizures (Table A1), and the data from these was thus used for the intra-subject evaluation, as well as the training set for the leave-one-participant-out cross-validation in the inter-subject evaluation. The remaining six participants had either one or two seizures recorded, for a total of eight seizures included in the inter-subject evaluation test set. The mean recording length per participant in this data set was 83.2 h (range 35.8–127.6 h).

**Figure 3:**
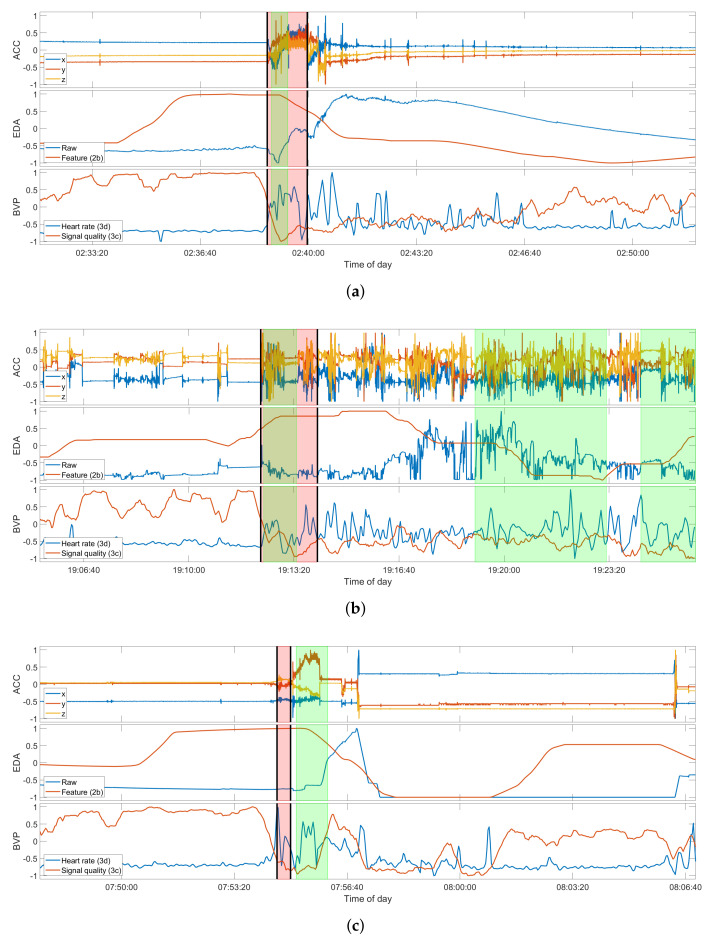
Selection of examples of true positive detections for each of the three participants in the intra-subject evaluation. Seizures shown are: (**a**) UKF1-4; (**b**) UKF2-3; (**c**) and KCL1-3 (see Table A1). Due to the grace period of 2 minutes around a seizure event, the detection for KCL1-3 counts as a true positive. Each plot of a seizure shows the raw ACC signal (top), the raw EDA signal and feature 2b (middle), and the estimated heart rate and signal quality index of the BVP signal (bottom). The regions highlighted in red mark the ground truth as labeled by experts, and those highlighted in green mark the seizure intervals, as predicted by the respective model, trained on the data of all the other seizures of the participant. The seizure onset and offset are additionally marked by the black vertical bars. All signals shown are normalized between −1 to 1 only for these plots. The original value ranges before normalization can be found in Table A3.

To give a better overview of the three participants with multiple seizures recorded and used in the intra-subject evaluation, Figure 3 presents one example seizure for each of these participants. Participant UKF1 had six seizures recorded with the wearable device, all of them focal onset motor seizures with tonic and clonic manifestations, ictal tachycardia, and impaired awareness. Furthermore, all of these seizures occurred while the patient was sleeping in his hospital bed, and all have a characteristic progression. Overall, these seizure symptoms the closest to focal to bilateral or generalized tonic–clonic seizures in the data set, yet noticeably lack the severity of the larger seizures, both regarding the vEEG and also the movements captured with the ACC signal. UKF2, on the other hand, had three focal onset motor seizures recorded with only tonic manifestations, ictal tachycardia, and miscellaneous awareness during the seizure. Notably though, all seizures occurred while the patient was awake. Another important distinguishing factor for this participant is that he was only nine years old at the time of enrolment, and as such the only pediatric patient in the relevant data sets regarded here. Epilepsy in pediatric patients generally manifests in different ways than for adults. The sole data set from the KCL site, KCL1, had three seizures recorded that match the criteria for the seizure type. The motor manifestations for them were more heterogeneous than for the other two participants. All had tonic components, but there were also some oral automatisms, and one seizure also had clonic components. Furthermore, one seizure did not prompt ictal tachycardia, and there was a high variance between the seizure durations, with one being over two minutes and occurring while awake, and the other two only 22 s, occurring from sleep. Aside from the movements during the seizures, Figure 3 also gives a good overview of the typical EDA and BVP responses in the data, which can be observed most clearly in the first presented seizure UKF1-4. The EDA signal shows a clear response to the seizure, and the feature, the difference in the maximum of the skin conductance level, accordingly, is at its highest during the seizure. The heart rate estimated from the BVP sensor signal also clearly demonstrates some response after the seizure onset for all three examples; however, at the same time, the signal quality also drops significantly, and as such the estimated heart rate should not be regarded as representative for these periods.

### 3.2. Intra-Subject Evaluation

Data from three participants were selected for the intra-subject evaluation. One patient was selected from the London cohort with three seizures recorded (KCL1), and two from the Freiburg cohort with six (UKF1) and three (UKF2) seizures recorded, respectively. Out of these twelve seizures, only one seizure, for participant UKF2, could not be identified in the individual optimized leave-one-seizure-out evaluation. All other seizures were consistently detected in the complete participant data by the optimized models, when trained on the other seizures for the respective participant. In terms of false alarm rate however, the methodology exhibited vastly different performances over the three participants. The cross-validation runs for participant UKF1 showed the lowest number of false positives at just three on average, ranging from 1 to 5 depending on which of the seizures was left out for testing. Overall, this results in a low false alarm rate of less than one per 24 h (0.85/24 h). For the other two cases, the false alarm rate was considerably higher, at almost two per hour for UKF2 (41.5/24 h) and somewhat less than one per hour for KCL1 (17.7/24 h), on average. An overview of the per-participant results of this evaluation can be found in Table 1 under “Intra-Subject Evaluation”.

### 3.3. Inter-Subject Evaluation

To assess the performance of seizure detection across multiple patients, the GTBM model is first trained using the peri-ictal seizure data of the 12 seizures from the three participants mentioned above. The model is thereby parameter-optimized in a leave-one-participant-out manner, as explained in the Materials and Methods. In this cross-validation, the model with the best-performing parameter combination was able to recognize a total of eight of the twelve seizures (overall sensitivity 67%, mean 72%, and range 50–100%) in the validation set, with a mean false alarm rate of approximately one per hour, averaged over the three participants (mean 24.4/24 h; range 0.5/24 h–64.9/24 h). The resulting model is then applied to the complete data sets of six other participants, including a total of eight epileptic focal motor seizures. In this out-of-sample test set, the model was overall able to detect six of the eight seizures (overall sensitivity 75%, mean 75%, and range 0–100%) with a mean false alarm rate of 13.4 per 24 h (range 4.4/24 h–22.7/24 h). Table 1 shows a summary of these across-participant results under “Inter-Subject Evaluation”.

### 3.4. Feature Importance

**Figure 4:**
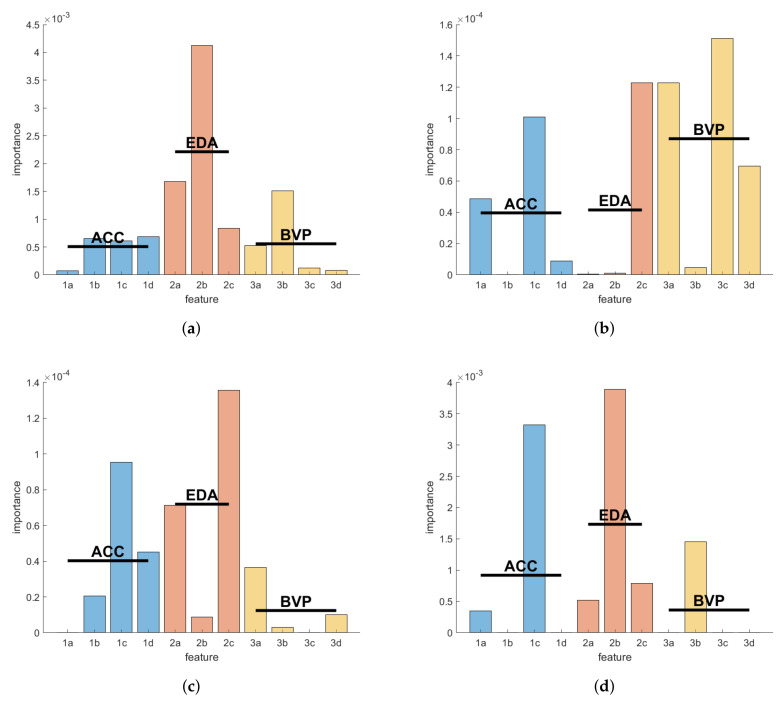
Feature importance scores per intra-subject evaluation for the seizure detection models of the three selected participants: (**a**) UKF1; (**b**) UKF2; (**c**) KCL1 (see Table A2); (**d**) Feature importance scores of the model resulting from training the GTBM model on the seizure data of all three inter-subject training participants. Blue, red, and yellow bars show the importance scores for the features grouped by biosignal modality ACC, EDA, and BVP, respectively. Horizontal lines mark the mean scores of the groups. The ordinate is unitless; the scores can be interpreted qualitatively. The feature labels correspond to the listing of features in the Materials and Methods.

The feature importance for each of the three optimal models trained on data from the three per-subject evaluation participants was calculated as outlined in the Materials and Methods. Figure 4 shows these importance scores per participant and feature, and the mean scores of each feature group by modality. These feature scores are unitless and can be interpreted qualitatively to determine whether some specific feature or general modality is contributing more than the others. Here, the EDA features were more influential than the others in both the participants, UKF1 and KCL1. Conversely, the BVP features were unexpectedly more meaningful for the model of participant UKF2 than the other two modalities. The same kind of feature importance scores for the inter-subject model trained on all three of these participants for the inter-subject evaluation can be found in Figure 4d. Here, the EDA features are demonstrated to be more important than the others.

## 4. Discussion

### 4.1. Principal Findings

The main ambition of the evaluation presented here was to qualitatively assess the utility of multimodal biosignal data from wearables in creating worthwhile and robust seizure detection systems. Thereby, two principal avenues of potential study design were investigated: intra-subject and inter-subject schemes. Specifically, we focused our evaluation on focal motor seizures with tonic or clonic components, as opposed to bilateral tonic–clonic seizures. These focal seizures have a multitude of possible physical and psychological manifestations that can occur in sequence or in parallel and be repeated or not occur at all, in a single seizure. Furthermore, while there may oftentimes be little change in the semiology of seizures for a single patient with epilepsy, they can be very heterogeneous across populations [2,44]. These circumstances are also reflected in our results. Among the three participants with at least three seizures recorded, the individually optimized model could robustly recover the left-out seizures in the leave-one-seizure-out cross-validation for two participants.

**Figure 5:**
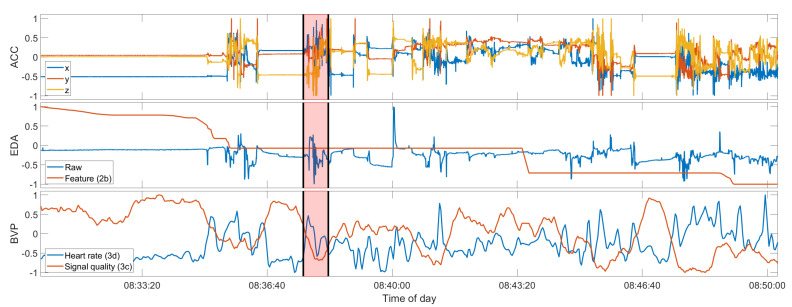
Seizure UKF2-2, a false negative. Compare also to Figure 3. Data shown from top to bottom: raw ACC, raw EDA and feature 2b, heart rate and BVP signal quality index. The red overlay is the seizure ground truth. The seizure onset and offset are additionally marked by the black vertical bars. All signals shown are normalized between −1 to 1 only for these plots. The original value ranges before normalization can be found in Table A3.

In one other participant, however, out of three seizures, one could not be restored by the model when trained on the other two (Table A1 and Figure 5). These three seizures had roughly the same semiology with tonic manifestations and ictal tachycardia. In the wearable data, however, one clear difference can be found between this seizure and the other two; that is, it had no discernable EDA response before, during, or after the seizure. Additionally, this participant UKF2 had an important demographic difference to all other included participants in this data set, in that they were the only pediatric patient at nine years old. Age has been linked, for example, to significant changes in seizure semiologies [45]. These circumstances likely led to this specific seizure falling out of the scope of this methodology.

These results suggest that a methodology such as the one presented here, optimized on individual participants, can robustly detect seizures for some patients with epilepsy, but it may fail, especially when the seizures have differing semiologies that are not represented in the training data for the model. Furthermore, when looking at the false alarm rate per 24 h (FAR24) and positive predictive values (PPV), the heterogeneity of focal seizure detection is especially highlighted. The FAR24 performance of the seizure detection, ranging from less than one false positive (FP) per day to almost two FP per hour, is an important factor when it comes to actually applying the methodology to a real-world setting. Similarly, this false alarm rate also carries over to the nighttime, with multiple false positives per night for some participants. Thus, a high sensitivity in detecting seizures is in vain if an automated seizure diary is filled with dozens of false seizure events per day. Yet, further data recordings and model optimization may produce robust seizure detection systems for individual patients.

With respect to the inter-subject evaluation across multiple study participants, the results for the methodology applied here further demonstrate the heterogeneity of the focal motor seizures in this data set, and clearly demonstrate the resulting difficulties. Inter-subject models applied in a leave-one-participant-out manner to data of the three selected participants from the intra-subject evaluation perform worse than if trained in an individualized manner, at least either in terms of sensitivity or false alarm rates. Likewise, testing the model on out-of-sample data of six other participants resulted in a tolerable sensitivity but a high false alarm rate and low PPV. A model such as this would be ineffective in real-world settings, be it as an automated seizure diary or an alarm system, and patients’ and caregivers’ needs, in particular, would not be fulfilled [46,47,48].

Overall, the results so far suggest that not only are focal onset motor seizures varied in their clinical manifestations, they are also sensitive to changes in common wearable biosignal modalities, when investigated across patients. However, in some individual patients, seizure semiologies are similar enough across seizures to enable robust seizure detection models for less-severe focal onset motor seizures, if the models are optimized in a personalized manner.

### 4.2. Related Work

To compare our results to some of the current and past state-of-the-art seizure detection studies, we compiled a list of twelve works featuring focal motor seizures in some form in their set of analyzed seizure types (Table 2). The main source of this list was the extensive literature review by Beniczky et al., 2021 [4], filtered for relevant seizure types and relevant biosignal modalities that are most closely related to the modalities ACC, EDA, and PPG. Furthermore, to add some variety, we also included two recent studies employing wearable EMG- and EEG-based focal seizure detection. We aimed to compare all three of our performance measures, namely sensitivity, FAR24, and PPV; however, as most of these works include focal motor seizures as part of a larger group of seizure types, almost always dominated by generalized and focal to bilateral seizures, we were only able to find three studies where this was possible for all three measures [9,11,20]. All three of these studies classify the focal onset seizures in their respective data sets as complex partial seizures (CPS), which is an older type of epileptic seizure classification meaning an interval with ictal impaired awareness without giving information about motor manifestations during the seizures. Therefore it is unclear if, with respect to movements during the seizures, the seizure types investigated in these works are comparable to those in the study presented here. Moreover, all three studies evaluated their seizure detection in an inter-subject manner across a population of patients with epilepsy.

Summarizing the relevant results from these three works, Cogan et al. 2017 [9] use a combination of two different wearable devices to record the biosignal modalities EDA, ECG, and SpO2, and report an algorithm sensitivity of 50% with a false alarm rate of 0.28 per day, in CPS only. Notably, during their analysis they also look into personalization of their algorithm, and conclude that a minimum of 6 to 8 seizures per patient would be required to sufficiently train and optimize their algorithm parameters, based on a worst-case scenario. Kusmakar et al. 2019 [11] employ ACC sensors in a leave-one-participant-out inter-subject evaluation and report a sensitivity of 67% and a FAR24 of 4, regarding the participants with CPS. They duly conclude that these seizures are much more similar to inter-ictal data than they are to ictal GTCS data, and as their data set includes only a very small number of CPS, a good performance on this seizure type is not to be expected. Lastly, Vandecasteele et al. 2017 [20] compared two wearable devices recording ECG and PPG, respectively, and reported an overall sensitivity of 32% with a FAR24 of 43.2 for the PPG-based wearable device, which was the same as the one used in this study. They conclude that the PPG-based detection was significantly hindered by motion artifacts even for these possibly non-generalized seizures.

Comparing these performances, and further results from other related work, which did not report their outcomes per seizure type or per participant (Table 2), in our results (Table 1), it becomes clear that a sensitivity of 75% in an independent test set of focal motor seizures is in fact among the top performing methodologies regarding this seizure type only. Furthermore, works that reported lower numbers of false positive rates also consistently had lower sensitivities, and some studies that reported similar numbers nevertheless still had lower sensitivities. Overall, a comparison of our results to any of these works should be taken with caution, as it is to the best of our knowledge the first work analyzing specifically focal motor seizures with multi-modal non-EEG wearable data.

### 4.3. Modality Importance

Overall, the distributions of feature importance scores, as a proxy for the importance of biosignal modalities, seem to be heterogeneous and no clear winner can be found amongst the three examples. The features calculated from the EDA signal, however, seem to be the most informative with respect to epileptic seizure phases, within this data set of focal onset motor seizures. This is concurrent with some prior research on generalized seizures as well [52,53,54,55], and suggests that electrodermal activity could be an important clinical marker of epileptic seizures beyond highly convulsive episodes, which usually induce heavy sweating. However, it is not a universally applicable biomarker, as these results also suggest, with at least one participant and several seizures exhibiting no significant (post-)ictal EDA response, as is also concluded in further literature on the topic [34,56,57].

In Glasstetter et al., 2021 [35], the authors explore the utility of wearable PPG signals for the detection of focal onset seizures with ictal tachycardia, and conclude that in some patients the tachycardia thresholds can be found regardless of seizure-related movements. It seems to be the case that in some seizures the ictal tachycardia presents itself some few seconds before the electrographic seizure onset, and could therefore be used as indicators for seizures, albeit not effectively in a monomodal system. The authors, however, also note that arbitrary non-seizure-related movements may hinder this detection from PPG signals due to motion artifacts, and generally, pre-ictal tachycardia seems to be an isolated phenomenon not generalizable over patient cohorts. Our results, demonstrating that BVP features are important only for one of three patients with at least three seizures recorded, coincide with these conclusions.

### 4.4. Limitations

The main limitation of the study and analysis presented here is the limited size of the data set, with just 20 seizures recorded from nine patients with epilepsy. Unfortunately, publicly available data sets focusing on classical wrist-worn wearable data which combine movement and autonomous nervous system biosignals are scarce as it is, and practically nonexistent in the field of epilepsy research. To be considered a useful supplement to the data collected here, a public data set would need to provide at least ACC, EDA, and BVP data recorded from a wrist-worn wearable in a cohort of patients with epilepsy, and at least EEG seizure onset and offset would need to be labeled by experts. To the best of our knowledge, such a data set does not currently exist in an open source form. The relatively small size of the data set used here is also the main reason we used “classical” supervised machine learning with a set of specifically tailored features as opposed to some deep learning methodology. Deep learning may, however, be an avenue to pursue in a later study, with a more extensive data set.

As the seizure types regarded here were carefully chosen, a large percentage of the overall recorded seizures were not eligible for inclusion. Furthermore, during their usually week-long stay at the epilepsy monitoring units, patients rarely have more than a few seizures in the first place, as seizure provocation is often conducted only for a limited time until enough seizures have been observed to serve some clinical purpose. This leads to a generally low number of study participants with more than two seizures recorded, a necessary minimum requirement for any kind of analysis of personalized, intra-subject evaluations. Future research may include more seizures and seizure types from our already recorded data set, but new studies need to be conceptualized to aim for higher numbers of seizures recorded per participant, not just over the whole cohort, in order to push focal seizure detection research to produce better, more realistically applicable results [46].

Another limitation of this study, as well as many other related works, is the confinement of participants to a hospital room throughout the data recording procedures. The data collected and analyzed here cannot be regarded as representative of real-world situations, and new ambulatory studies with a prospective goal of recording focal onset seizures with wearables from patients in their daily living environment are needed. However, even with generalized tonic–clonic seizures as a target, these phase 3 and 4 studies are still rare, and potential methodologies and pitfalls need first be explored in these in-hospital studies before worthwhile out-of-hospital studies can be designed.

## 5. Conclusions

Seizure detection for focal onset seizures without generalization by means of wearable non-EEG devices is a so far little-researched problem. We demonstrate that for those seizures in this category with tonic or clonic movements, that is, those closest in semiology to generalized tonic–clonic seizures, robustly detecting seizures from wearable data may be possible for individual patients with epilepsy, depending on their specific seizure manifestation. Overall, electrodermal activity signals seem to provide the most informative features for seizure detection, suggesting it to be an essential part of any future seizure detection research. Detection across patients with purely inter-subject models without personalization is, however, not possible to a worthwhile degree, at least with the methodology and data set presented here. Both a low sensitivity that misses a quarter or more of the seizures and high false alarm rates in the order of one per hour make current results of these inter-subject models ineffective for clinical applications. Our results thus demonstrate that individualized models are needed to robustly detect focal onset seizures, and future research in this domain should include at least some degree of personalization in the modeling process.

## Figures and Tables

**Table 1:** Evaluation results for the intra-subject leave-one-seizure-out evaluation, and the inter-subject leave-one-participant-out evaluation, respectively. Means and ranges are always across the single folds of the validations, that is, across the held-back data for the first part and across the held-back data, and test set participants, in the second part. FP: false positive; FAR24: false alarm rate per 24 h; PPV: positive predictive value; FAR: false alarm rate; LOPO: leave-one-patient-out cross-validation.

Patient ID	Sensitivity	Mean FP [Range]	Mean FAR24 [Range]	Mean PPV [Range]	Mean FAR per Night [Range]	Recording Duration	Device on Same Hand as Seizure
**Intra-Subject Evaluation**							
UKF1	100% (6/6)	3 [1–5]	0.85 [0.28–1.42]	28.3% [16.7–50%]	0	84.4 h	100% (6/6)
UKF2	67% (2/3)	79 [18–126]	41.52 [9.42–65.94]	0.6% [0–1.1%]	6.3 [1–11.5]	45.9 h	100% (3/3)
KCL1	100% (3/3)	37 [0–58]	17.69 [0–27.72]	34.5% [1.7–100%]	1.9 [0–3.0]	50.2 h	0% (0/3)
**Inter-Subject Evaluation**							
LOPO UKF1	50% (3/6)	28	7.96	9.7%	3.1	84.4 h	100% (6/6)
LOPO UKF2	100% (3/3)	124	64.9	2.4%	9.5	45.9 h	100% (3/3)
LOPO KCL1	67% (2/3)	1	0.48	67%	0	50.2 h	0% (0/3)
LOPO test (N = 6)	75% (6/8)	55 [16–87]	13.4 [4.4–22.7]	2.1% [0–5.9%]	2.0 [0.7–3.2]	568.6 h	38% (3/8)

**Table 2:** Related work compiled from Beniczky et al., 2021 [4], and this study as comparison. Only those works are included that involve seizure types relevant to this study, that is, any of focal motor seizures, SPS, CPS, or other non-generalized seizures. FAR24: false alarm rate per 24 h; PPV: positive predictive value; FS: focal seizures; SPS: simple partial seizures; CPS: complex partial seizures; hyper: hypermotoric seizures; myo: myoclonic seizures; FS min mot: focal seizures with minimal motor component.

Study	Modalities	Seizure Types	# Pat. w/Seizures	# Seizures	Sensitivity	FAR24	PPV
*this study*	*ACC, EDA, PPG*	*FS tonic or clonic*	*9*	*20*	*67–100%/75%*	*0.85–41.5/13.4*	*0.6–34.5%/2.1%*
[6] ^+^	ACC, PPG	FS hyper/other major	(28 total)	5/14	73%/84%	Not reported per seizure type	
[8] ^+^	ECG	FS/SPS/CPS/other	(31 total)	8/26/31/5	Not reported per seizure type		
[9] ^+,^*^,§^	EDA, ECG, SpO2	CPS	8	23	16.7%/50%	0.7/0.28	6.25%/50%
[10] ^+^	ECG	SPS/CPS	(16 total)	37/38	19%/71%	Not reported per seizure type	
[18]	ACC	Tonic/tonic-clonic	15	22	67%/100%	Not reported per seizure type	
[11] ^+,^*	ACC	CPS	3	5	67%	4.19	22.5
[49] ^+^	ACC, EDA	FS tonic-clonic	2	6	50%	Not reported per seizure type	
[12] ^+^	ACC	Myo, tonic/FS hyper/FS min mot	(41 total)	140	6%/24%/2%	Not reported per seizure type	
[14] ^+^	ACC, ECG	FS hyper/myo, tonic cluster	5/5	18/9	Not reported per seizure type		
[20]	ECG/PPG	CPS	11	47	70%/32%	50.6/43.2	2.15%/1.12%
[50]	EMG	GTCS/tonic/clonic/ other motor	20	18/9/3/17	83%/56%/33%/76%	-	83%/50% (t+c)/76%
[51]	EEG, ECG, ACC	Focal tonic/focal nonmotor	3	47/9 + 9	84%/100%	8/13 + 5	-

^+^ The study also contained other seizure types, most notably generalized seizures, however the presented data only relates to those seizure types specifically mentioned. * Performance scores only include CPS, calculated by authors from original reported numbers. ^§^ Performance scores represent a non-optimized detection, and a refined analysis, respectively.

## Data Availability

Not applicable.

## Appendix A

**Table A1:** Seizures recorded for the three participants used in the intra-subject evaluation. iTC: ictal tachycardia; UI: urinary incontinence.

Seizure ID	Seizure Duration	Motor Symptoms	Autonomic Symptoms	Awareness	Vigilance/Body Position
UKF1-1	82 s	Tonic, clonic	iTC	Impaired	Asleep/lying
UKF1-2	86 s	Tonic, clonic, myoclonic, automatisms (arms, legs)	iTC, UI	Impaired	Asleep/lying
UKF1-3	55 s	Tonic, clonic, myoclonic	iTC	Impaired	Asleep/lying
UKF1-4	73 s	Tonic, clonic, myoclonic, automatisms (legs)	iTC	Impaired	Asleep/lying
UKF1-5	43 s	Tonic, clonic	iTC	Impaired	Asleep/lying
UKF1-6	47 s	Tonic, clonic, myoclonic	iTC	Impaired	Asleep/lying
UKF2-1	23 s	Tonic	iTC	Aware	Awake/sitting
UKF2-2 *	39 s	Tonic	iTC, flushing	Impaired	Awake/lying
UKF2-3	107 s	Tonic	iTC, flushing	Impaired	Awake/sitting
KCL1-1	128 s	Tonic, clonic, automatisms (arms, face)	iTC	Impaired	Awake/sitting
KCL1-2	22 s	Tonic, automatisms (face)	iTC	Impaired	Asleep/lying
KCL1-3	22 s	Tonic, automatisms (face)	-	Impaired	Asleep/lying

* Seizure was not recognized by the model during evaluation.

**Table A2:** Demographic and clinical information for the nine selected participants. TLE: temporal lobe epilepsy; FLE: frontal lobe epilepsy; xTLE: extratemporal lobe epilepsy.

ID	Gender	Age	Total Recording Duration	# Seizures Recorded	Epilepsy Origin	Epilepsy Type
UKF1	m	55	84.4 h	6	Structural	Focal (TLE)
UKF2	m	9	45.9 h	3	Structural	Focal (xTLE)
UKF3	f	27	92.0 h	2	Structural	Focal (TLE)
UKF4	f	69	120.8 h	1	Structural	Focal (TLE)
UKF5	m	50	127.6 h	2	Structural	Focal (FLE)
UKF6	f	34	35.8 h	1	Unknown	Focal (FLE)
UKF7	m	48	105.1 h	1	Structural	Focal (TLE)
UKF8	f	46	87.2 h	1	Structural	Focal (TLE)
KCL1	m	65	50.2 h	3	Structural	Focal (TLE)

**Table A3:** The original value ranges for the plotted data in Figure 3 and Figure 5, before normalization. ACC: accelerometry; EDA: electrodermal activity; BVP: blood volume pulse; HR: heart rate.

Seizure ID	Data Range		ACC x [g]	ACC y [g]	ACC z [g]	EDA Raw [μs]	EDA Feature [a.u.]	BVP HR [bpm]	BVP Quality [a.u.]
UKF1-4	ictal	min	−1.7031	−0.9688	−0.6406	0.2602	5.0397	50.7644	0.0917
		max	0.6094	0.2344	1.9844	5.7441	7.8344	121.0513	0.2325
	non-ictal	min	−1.1563	−1.3750	−0.5313	2.0375	−4.4675	48.9167	0.1241
		max	0.9219	0.0625	1.1875	11.1237	8.0093	137.2188	0.3376
UKF2-2	ictal	min	−1.5000	−1.1406	−2.0000	0.2770	−0.0152	70.0432	0.1664
		max	1.8906	1.9844	1.0938	0.6421	−0.0152	102.9488	0.2530
	non-ictal	min	−2.0000	−2.0000	−2.0000	0.2975	−0.0385	57.6646	0.1367
		max	1.9844	1.9844	1.9844	0.8484	0.0117	119.5292	0.32395
UKF2-3	ictal	min	−2.0000	−1.2500	−2.0000	0.0051	0.1558	51.2767	0.1242
		max	1.4219	1.7031	1.9844	0.3472	0.2559	124.4696	0.2306
	non-ictal	min	−2.0000	−2.0000	−2.0000	0.0000	−0.2570	43.3582	0.1185
		max	1.9844	1.9844	1.9844	0.6789	0.2959	147.5059	0.312448
KCL1-3	ictal	min	−0.9219	0.2969	0.3281	5.9464	33.3491	66.5748	0.1346
		max	−0.7188	0.4219	0.6875	6.3181	33.5858	136.6889	0.1641
	non-ictal	min	−1.4688	0.0469	−1.4688	0.0000	−40.6902	54.5837	0.1157
		max	0.9063	0.6875	1.9063	51.4717	33.7534	118.9363	0.3457851
